# Substantial Increases in Healthcare Students’ State Empathy Scores Owing to Participation in a Single Improvisation Session

**DOI:** 10.3390/ijerph21050531

**Published:** 2024-04-25

**Authors:** Brian D. Schwartz, Shane L. Rogers, Nicole Michels, Lon J. Van Winkle

**Affiliations:** 1 Department of Medical Humanities, Rocky Vista University, Englewood, CO 80112, USA; bschwartz@rvu.edu (B.D.S.); nmichels@rvu.edu (N.M.); 2 School of Arts and Humanities, Edith Cowan University, Joondalup, WA 6027, Australia; shane.rogers@ecu.edu.au; 3 Department of Biochemistry, Midwestern University, Downers Grove, IL 60515, USA

**Keywords:** compassion, empathy, healthcare professionals, improvisation, questionnaire validity, patient care, patient satisfaction, survey reliability

## Abstract

Purpose: To determine whether the 12-item state empathy scale could be modified reliably to measure empathy in healthcare professions students and to detect changes in their empathy owing to a single improvisation (improv) session. Methods: Three cohorts of students from two healthcare professions programs (total = 165 students) participated in an improv session. During the session, one of the researchers (BS) tasked the students with several improv activities. Participants’ self-reported state empathy scores were assessed at three time points (pre-improv, post-improv, and end of semester) using revised, in-class paper versions of the State Empathy Scale. Results: The exploratory factor analysis revealed a single factor solution for the revised scale, justifying the creation of an overall state empathy score from the questionnaire. Cronbach’s alpha reliability values averaged 0.87. Students’ mean empathy scores were higher directly after the improv session than directly prior to the session (*p* < 0.0001; effect size = *r* = 0.67, 0.55, and 0.79 for cohorts 1, 2, and 3, respectively). Conclusions: These findings show that a single one- or two-hour improv session can foster substantial increases in healthcare professional students’ state empathy for one another. Greater healthcare professional empathy and compassion foster better healthcare team cooperation and patient outcomes, so healthcare professionals and their students should engage in such empathy-enhancing activities at regular intervals throughout their training and careers.

## 1. Introduction

Higher healthcare provider empathy and compassion improve the quality of patient care and decrease the probability of practitioner burnout [1,2]. For example, practitioner empathy reduces the risk of serious complications in diabetic patients. When such conditions become life-threatening, provider compassion decreases the rate at which patients experience post-traumatic stress disorder. For these reasons and more [1,2], a continuing goal in healthcare professions training should be to foster empathy and compassion in these students [3,4]. 

According to Hojat [5], compassion is where affective and cognitive empathy overlap. In healthcare, a provider should use affective empathy (i.e., sympathy) to sense patients’ feelings to some extent [6], while using cognitive empathy to “understand the kind and quality of patients’ experiences” [5] (chapter 1, p. 12). That is, providers should “feel patients’ pain” but not in a way that impairs “understanding their suffering”. Thus, scores on reliable and valid surveys known to measure affective and cognitive empathy show considerable correlations between these measures of empathy [7] (e.g., *r* = 0.45). Accordingly, the State Empathy Scale used in the current study includes dimensions of both affective and cognitive empathy, as well a third dimension known as associative empathy (i.e., relationship development) [8].

### 1.1. Teaching/Fostering Empathy in Healthcare

Zhang et al. [9] reviewed how medical humanities courses fostered empathy in medical students—according to quantitative measures—particularly when the courses required reflective writing by students. When reflective writing was not required [10,11], successful efforts at increasing students’ empathy involved narrative medicine-based education and reflective practice Balint groups [12]. Similarly, empathy was fostered in prospective medical students [4] and, prior to these studies, in medical students [3]. In all of the latter studies [3,4], students’ empathy was fostered via writing critical reflections about their team service-learning projects.

A broader review of empathy-inducing teaching strategies was published recently by Silva and associates [13]. These empathy teaching strategies include, but are not limited to, team discussions, artistic productions, simulations, storytelling, narrative medicine, ethical dilemmas, the expression of feelings and emotions, brainstorming, and communication to understand the patient’s point of view. While qualitative results support the conclusion that many of these teaching strategies are successful, it has not been determined whether the strategies foster higher provider empathy, as measured by reliable and validated quantitative surveys. Additional teaching strategies that have received some interest are improvisation activities [14,15,16].

### 1.2. Actors, Improv, and Empathy

Improvisation skills can be central to the success of actors and the fostering of their empathy for one another [17]. In this regard, Schmidt and associates [18] found that prospective actors expressed above average empathic concern and a high ability to recognize emotions while not feeling great distress. However, higher education psychology students scored at least as high or higher on measures of empathy than did acting and dance students. It would be interesting to learn whether the empathy scores of these various groups of students change differently during their training.

### 1.3. Improvisational Exercises to Increase Empathy and Compassion

O’Connell et al. [16] used improvisational exercises at the Stony Brook University Alan Alda Center for Communicating Science to build empathy between university mentors and their student mentees majoring in sciences in graduate-level programs. While they collected opinions about their workshop from faculty and students, these authors did not employ reliable and validated surveys to measure changes in participants’ empathy and compassion. Similarly, Kaplan-Liss and associates [14] designed an interprofessional elective course for health professions students at the Alda Center using improvisation to enhance empathetic communication, but, again, only opinions about the course were collected from students.

In another elective improv course for health professions students, however, some quantitative evidence supported the conclusion that students’ empathy increased owing to a six- to eight-week, six-session course (2.5 h per session) [15]. The mean scores of the 45 students enrolled in the course increased significantly for half of the 10 items on a self-report version of the Consultive and Relational Empathy (CARE) measure—namely the ease, care, explain, help, and plan items. However, no improvement was detected by any item on the Interpersonal Reactivity Index (IRI), another self-report empathy measure.

In Zelenski et al.’s study [15], numerous other activities were included in sessions in addition to improv exercises. These other activities included discussions about pertinent readings and experiences, debriefings after improv games, the consideration of connections to team interactions in clinics, and, at the end of the class, five minutes of free writing about students’ experiences in class that day [15]. In this regard, though, more activities may not necessarily be better when it comes to improving quantitative and self-reported student empathy scores in improv courses. For example, it has been reported that shorter humanities course interventions can be equally as beneficial as longer ones [9]. Consequently, it seems possible that including other activities in improv courses, as others have done [14,15,16], may detract/distract healthcare professions students from the improv exercises themselves. It also makes it difficult to fully understand whether improv activities impact empathy beyond the other activities being undertaken. Consequently, the focus here was the effect of improv exercises on students’ state empathy directly before versus directly after they participated in these exercises and without including any other activities in the improv sessions themselves.

### 1.4. Approach Used to Test Whether Improvisation Can Foster Empathy in Health Professions Students

A single one- or two-hour improvisation session was used to attempt to foster empathy in health professions students. The session included only improv exercises themselves. The success of the session was measured using modifications of the State Empathy Scale developed by Shen [8]. Since the scale was modified considerably, it was also determined whether the modified surveys were reliable. The three hypotheses were as follows.

○ Hypothesis one: The revised State Empathy Scales are reliable measures of healthcare professions students’ empathy.
○ Hypothesis two: An improvisation (improv) session will immediately result in higher state empathy scores among healthcare professions students.
○ Hypothesis three: An improv session will foster students’ interprofessional state empathy.

## 2. Methods

### 2.1. Participants and Procedure

Participants were matriculants at the Colorado campus of Rocky Vista University. There were three cohorts of all first-year Master of Science in Biomedical Sciences (MSBS) students during the fall semesters of 2018, 2019, and 2023, joined by all first-year Physician Assistant (PA) students in 2018 and 2019. That is, students in the two programs worked together in small groups in a single improv session in 2018 (cohort one) and 2019 (cohort two), whereas only MSBS students participated in the improv session in 2023 (cohort three). Ninety-two percent of MSBS students graduate with a Master of Science degree and gain admission to medical school. All of these MSBS and PA students were selected for this study because they were enrolled in courses in which an improv session was scheduled.

The one- or two-hour improv sessions, conducted by a faculty member with improv acting and comedy experience, consisted of guided, extemporaneous games and exercises, designed to engender communication, trust, presence, and active listening and participation. These activities included nothing scripted or predetermined and were unrehearsed and without discussion. The exercises included set frameworks within which the participants created their own dialogue, conversations, and actions.

The first exercise was a mirroring game in which participants arranged themselves in groups of roughly five and mimicked an action taken by one participant. Students were cautioned that the action needed to be imitated exactly, including not only the action itself but the expressions, stance, proxemics, and body language. The goal was to pay active and close attention to not only the actions taken but also the accompanying non-verbal communications, with the intention of active and total observation, and to focus on everything that the leader was conveying, both intended and unintended.

The second exercise was a paired storytelling game in which the facilitator gave an introductory story sentence and each pair had to create and tell the rest of the story, in alternating lines. The game was played in three rounds. In the first of these, each subsequent sentence had to start with the word “no”. The second had to start with “yes, but…”, and the third began with “yes, and…”. After an appropriate amount of time in each iteration, the faculty member asked for brief reflection and comments from the participants as to the nature of their story, specifically the storytelling arc. Students invariably noted that with each change in the opening of their responses, the arc was more linear, more productive from a narrative standpoint, less frustrating, and more engaging. This exercise is designed to communicate to healthcare professions students how much more conducive to hearing and communicating “yes, and” is than either “no” or “yes, but”.

Other exercises included “yes, let’s!”, in which all participants walked briskly but unguided throughout the classroom until one person yelled “let’s go to the _____”, with the blank being filled by a location or activity. Examples included the gym, a movie, a music concert, a yoga class, the anatomy lab, and many more. Upon hearing the destination, the rest of the students replied with “yes, let’s!” and immediately struck a pose or pantomime appropriate to that location or circumstance. As an example, for “the gym”, students adopted a posture of lifting weights, running on treadmills, playing pickleball, and swimming, among many others. 

The final improv game, entitled “Dr. Know-it-all”, was a game in which a team of five to seven students combined to become an “expert on everything in the world”. The team would go to the front of the classroom and random others would ask them a question, ranging from scientific in nature to silly and everything in between. The group members would then answer the question, extemporaneously, each saying only one word, to be combined into sentences and eventually form an answer to the question. 

Although not specifically medical in nature, each of these improv activities was intended to build good relationships among the healthcare professions students in general. Such relationships are essential to providing the best care to patients, as well as the most productive working relationships more broadly [14,15,16]. In this regard, the State Empathy Scale used in the current study included dimensions of affective, cognitive, and associative empathy, each of which fosters social bonding and relationship development [8].

### 2.2. Surveys and Experimental Design

With permission and encouragement from Dr. Shen, the original 12-item State Empathy Scale [8] was modified for completion by MSBS and PA students working together in the same improv session (cohorts one and two, Appendix A) or MSBS students working only with one another (cohort three, Appendix B). Since the scale was originally designed to measure audience responses to messages from a character, such as in a public health campaign, the scale was revised to assess healthcare professions students’ thoughts and feelings about their classmates. For example, item one on the original scale was modified from “The character’s emotions are genuine” to read, on the modified scale, “My classmates’ emotions are genuine” (Appendix A and Appendix B).

The difference between the two scales in the Appendices is in the instructions at the beginning of the surveys. For cohorts one and two, the instructions specify “students in the other program” on the survey form (underlined in Appendix A), whereas “students in the MSBS program” are specified on the survey form for cohort three (underlined in Appendix B). Students completed the surveys directly before the one- or two-hour improv session began and directly after it ended. In order to match students’ first and second survey results, they were randomly assigned a survey number for their anonymous paper surveys. To test whether changes in the students’ state empathy scores might be stable for more than one improv session, the students in the first two cohorts also completed the scale at the end of the semester in which the improv session was performed. The course in which students in the third cohort participated in improv lasted only for the first half of the semester, so they did not complete the survey at the end of that term. One of the authors (BS) collected the anonymous paper surveys and returned them to another author (LV) for data analysis.

The Rocky Vista University Institutional Review Board (IRB) found that this study (HIRB# 2018-0043) satisfied the criteria for exemption.

### 2.3. Statistical Analyses

GraphPad Prism 10.2.0 Software Inc. (La Jolla, CA, USA) was used to calculate Cronbach’s alpha values by employing two-way analyses of variance, which yielded mean square values and where Cronbach’s alpha = 1 − (residual mean square/row factor mean square). The Stata statistical program was used for survey item factor analysis [19]. 

Using the GraphPad software, the impact of the improv session on self-reported empathy was tested by measuring students’ mean scores on a revised State Empathy Scale directly before versus directly after the improv session and comparing these means statistically using paired *t*-tests. When the scale was administered a third time at the end of the semester (student cohorts 1 and 2), the mean scores were also compared statistically using one-way analysis of variance combined with multiple comparison tests of score values matched for each student. Please see Appendix C for the mean and standard deviation (SD) values calculated for each set of data collected directly before the improv session, directly after the improv session, and at the end of the semester in which the sessions took place. The latter mean and SD values were calculated independently of other sets of data.

## 3. Results

### 3.1. Response Rates

There were 63 students in cohort one, comprising 36 (57%) PA and 27 (43%) MSBS students. Of these 63 students, 58 (92%) completed the revised State Empathy survey. Similarly, cohort two was composed of 36 (51%) PA and 34 (49%) MSBS students. Of the latter 70 students, 65 (93%) completed the empathy survey. Finally, cohort three had 45 MSBS students, 42 (93%) of whom completed the survey.

**Hypothesis 1:**  *The revised State Empathy Scales are reliable measures of healthcare professions students’ empathy*.

The reliability of the empathy scales used in this study was tested via exploratory factor analysis. Exploratory factor analysis was conducted using the statistical program Stata [19]. More specifically, the default “principal factor” method was used in Stata, which analyzes the common variance, instead of the total variance, which is analyzed via principal components analysis. This approach was taken because the factor structure of the revised versions of the State Empathy Scale was being examined for the first time.

In the original version of the State Empathy Scale, Shen [8] provided data to suggest that the questionnaire could be broken down into three correlated sub-aspects. The factor analysis results in the present study indicate that a single factor is the most straightforward conceptualization of the measure; see Table 1. The Cronbach’s alpha reliability values for this overall empathy measure were found to be good across all cohorts and time points (ranging from 0.77 to 0.91; see Table 1).

**Hypothesis 2:**  *An improvisation (improv) session will immediately result in higher state empathy scores among healthcare professions students*.

#### 3.1.1. Third Cohort Comprising Only MSBS Students

**Figure 1:**
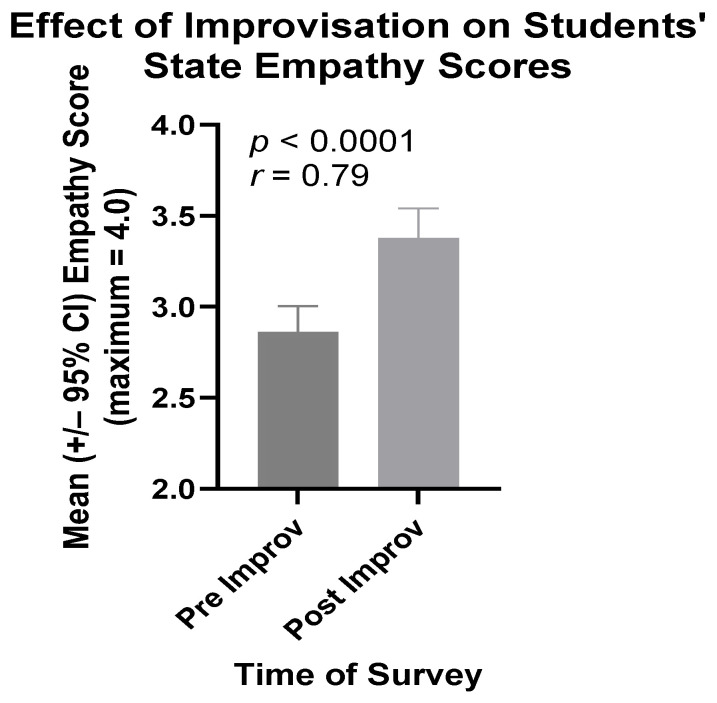
Mean State Empathy Scale scores of cohort three students directly before (Pre) and directly after (Post) participation in their improv session.

The mean state empathy scores of students in the third cohort rose in a highly significant manner between the beginning and the end of their improv session. As shown in Figure 1, this difference was both statistically significant (*p* < 0.0001) and of crucial practical importance (*r* = 0.79). That is, this effect size (*r* value) indicates the extent to which hypothesis two is true [20,21]. 

#### 3.1.2. Cohorts One and Two Each Comprising MSBS and PA Students Working Together

**Figure 2:**
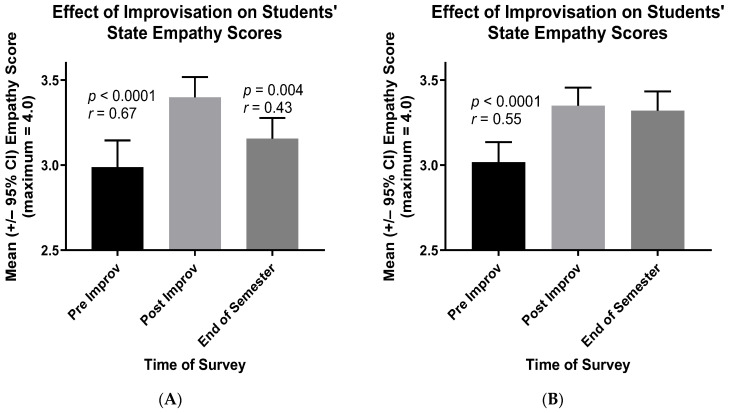
Mean State Empathy Scale scores of cohort one (**A**) and cohort two (**B**) students directly before (Pre) and directly after (Post) participation in their improv sessions. Also shown are students’ mean scores at the end of the semester in which they participated in their improv session.

Similarly to the students in the third cohort, the mean state empathy scores of the healthcare professions students in the first and second cohorts rose substantially between the beginning and end of their improv session (Figure 2). When students in the PA and MSBS programs worked together in teams of five to seven during improv, their mean state empathy scores increased in highly significant ways, with effect sizes (*r* values) [20,21] of crucial practical importance (Figure 2A, first cohort, *r* = 0.67, *p* < 0.0001; Figure 2B, second cohort, *r* = 0.55, *p* < 0.0001). The mean scores of students in the first cohort then declined by the end of the semester (Figure 2A, *r* = 0.43, *p* = 0.004), but such was not the case for students in the second cohort (Figure 2B).

**Hypothesis 3:**  *An improv session will foster students’ interprofessional state empathy*.

**Figure 3:**
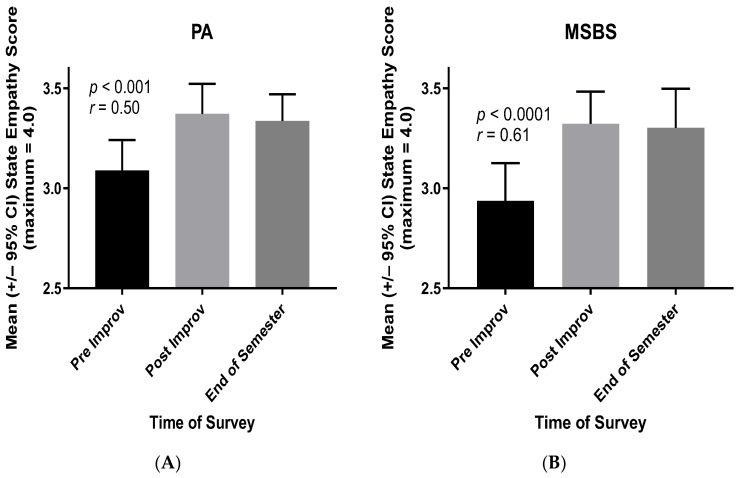
Mean State Empathy Scale scores of cohort two PA (**A**) and MSBS (**B**) students—regarding students in the other program (Appendix A)—directly before (Pre) and directly after (Post) participation in the improv session. Also shown are students’ mean scores at the end of the semester in which they participated in the improv session.

The latter results (Figure 2) imply that the gains in students’ empathy—owing to improv participation—extend not only to their classmates but also to students in the other healthcare professions program. That is, between Physician Assistant (PA) students and MSBS students, the vast majority of whom then gain acceptance to medical school. To verify this conclusion, the results for cohort two (Figure 2B) were separated into PA and MSBS students. As shown in Figure 3, both PA and MSBS students reported increased empathy for “students in the other program” following the improv session and as instructed in their revised State Empathy Scale (Appendix A). The effect sizes (*r* values) of these increases in empathy scores were of crucial practical importance [20,21].

## 4. Discussion

Three hypotheses were tested in this study. First, it was shown that revisions of the State Empathy Scale are reliable measures of healthcare professions students’ attitudes and feelings toward one another. Exploratory factor analyses revealed that a single overall empathy score was the most appropriate conceptualization of the revised State Empathy Scales used in this research. The Cronbach’s alpha values for student responses to the revised State Empathy Scales were “good” for each of the three cohorts of students and varied from 0.77 to 0.91 (mean alpha value = 0.87).

Hence, the revised scales could be used reliably to test whether the improv sessions led to changes in empathy scores among students in three separate cohorts. Significantly, the mean scores of the students in each cohort were greater directly after the improv session compared to directly prior to it (*p* < 0.0001), and these increases were of “crucial practical importance” (effect size = *r* = 0.67, 0.55, and 0.79 for cohorts 1, 2, and 3, respectively). To our knowledge, this is the first quantitative demonstration that a single improv session can profoundly increase healthcare professions students’ empathetic feelings and attitudes toward one another. For example, 40 of 42 students who completed the survey in cohort three demonstrated increases in their state empathy scores.

Finally, the gains in empathy owing to improv participation extended to students in another healthcare professions program. That is, PA students gained greater empathy for “students in the other (MSBS) program” and vice versa (Figure 3 and Appendix B). Moreover, MSBS students overwhelmingly gained acceptance to medical school following graduation from the MSBS program.

Such effects are important because students become practitioners who must work together effectively in interprofessional teams to maximize the quality of the healthcare that they deliver [22,23]. In some ways, though, this impact of improv on healthcare professions students may be more emotional than rational. For example, the size of the increase for items such as “I am in a similar emotional state as my classmates when dealing with them” was more than three times greater among all 165 students than the still significant increase (*r* = 0.24, *p* < 0.002) in items like “I can see my classmates’ points of view”. Moreover, healthcare providers might benefit from knowledge of such differences in their delivery of team healthcare. It also remains to be determined whether these increases in empathy extend to patients and their families as part of these teams.

To maintain this higher empathy, the incorporation of regular improv sessions throughout the curricula of healthcare professions students is recommended. As can be seen especially in Figure 2A, students’ empathy scores likely begin to decline after single interventions such as an improv session. In a prior study with a similar design, significant increases were observed in the empathy scores of pharmacy and medical students immediately after participation in a workshop on aging [24]. However, this increase was lost before the end of the academic terms of the latter students. In the case of pharmacy students, it took only seven days for this loss to occur [24]. When these students become practitioners, regular critical reflection on the challenges and rewards of their work and lives also will likely help them to maintain compassion and avoid burnout [1,2,3,4]. Healthcare providers often work on their own to sustain these efforts.

### 4.1. Health Professionals’ Strategies

Many healthcare professionals have developed and maintained their own strategies to foster empathy and compassion toward their patients. For example, in a sample of 151 physicians, nurses, psychologists, and other health professionals in New Zealand and elsewhere, 60% of respondents reported using self-focused strategies to maintain empathy and compassion toward their patients, while 38% used patient-centered approaches [25]. These categories of strategies were coded by the investigators according to practitioners’ responses to the question “…How do you maintain compassion for your patients inside yourself?” Samples of participants’ responses included “I try to imagine their situation from their perspective and put myself in their shoes” (i.e., empathy) and “I acknowledge that I am human and cannot fix everything” (i.e., self-care). In addition to their own strategies, healthcare professional trainees should be provided with a variety of empathy- and compassion-enhancing activities in their curricula.

### 4.2. Elements Influencing Execution and Maintenance of Compassion Andragogy

Sinclair and associates [26] describe a number of elements that impact the execution and maintenance of compassion andragogy. Most important to implementing and sustaining these programs is strong leadership by the senior administrators of the organizations attempting to start and sustain such components of their curricula for healthcare professionals and students [27]. In spite of evidence showing that compassion training fails to be sustained because of a lack of adequate leadership, most such programs focus on healthcare practitioners and not top leaders. This lack of adequate leadership likely leads to compassion training programs that are too short to maintain change [27].

### 4.3. If Provider Compassion Improves Patient Satisfaction and Care, Compassion Training Should Be an Integral Part of Healthcare Students’ Curricula

All of the factors that influence the implementation and maintenance of compassion training programs [26,27] also likely prevent their more permanent incorporation into curricula. Empathy is an important dimension of compassion, and empathy-enhancing teaching strategies include not only improv. They also involve team discussions, artistic productions, simulations, storytelling, narrative medicine, ethical dilemmas, the expression of feelings and emotions, brainstorming, and communication to understand the patient’s point of view [13]. However, the implementation of such training throughout a healthcare professional curriculum—with or without integration among courses—has not, to our knowledge, been attempted and reported. Additional barriers to such attempts also exist.

As preclinical faculty members, some of the present researchers have directly observed resistance to such efforts, which are considered unnecessarily distracting, especially to medical students, many of whom may feel that they have more important basic science course content to master. During clinical training, we contend that other aspects of this more or less “hidden curriculum” may block these efforts. Beyond the hidden curriculum, however, lie often ignored institutional and organizational systems that help to perpetuate undesirable cultural expectations.

For example, Martimianakis and associates point out [28] a preoccupation in the literature with the hidden curriculum as antagonistic to the idealistic humanism training needed to produce more compassionate medical providers. However, resultant efforts to foster empathy and compassion in practitioners usually target medical faculty and students, while largely ignoring how institutions and sociopolitical relations produce undesirable and unwanted practitioner behavior. The hidden curriculum can help to expose these system-level realities in the delivery of healthcare and, thus, reveal the gap dividing the real versus the ideal ways in which medicine is practiced [28]. Such realizations among healthcare providers and especially their students should help them to actively advocate for more just practices in the manner in which healthcare systems are organized and, in turn, how this care is delivered.

Since justice is one important goal in healthcare delivery, there are ways in which the social determinants of health (SDOH) should be taught. According to Sharma and associates [29], educators should not merely teach healthcare professional students about SODH. More importantly, they ought to promote students’ desire to take action against inequity in healthcare and, thus, to foster their commitment to social justice. To maintain this action and commitment, students also need to learn regularly to perform self-examination through critical reflection [3,4].

## 5. Limitations and Future Research

While the results of this study are encouraging, it remains to be determined whether patients perceive practitioners—who participate regularly in improv sessions—as more empathetic as determined with, say, the Consultive and Relational Empathy (CARE) measure [30]. Moreover, if patients do perceive higher empathy in such caregivers, it needs to be determined whether this higher empathy leads to better patient outcomes. While higher healthcare provider empathy and compassion clearly improve the quality of patient care [1,2], it remains to be determined whether the higher state empathy specifically associated with practitioner participation in improv sessions also improves care. Moreover, this investigation was performed at a single institution with limited sample sizes of only two types of healthcare professions students. While the findings were replicated on three independent occasions with highly significant statistical results, none of these replicates included a comparison control group. For all of these reasons, the conclusions regarding empathy changes owing to improv participation are provisional.

## 6. Conclusions

In the present study, quantitative data are reported to demonstrate that participation in improvisation activities—facilitated by a medical humanities faculty member and that aimed to foster empathy in healthcare professions students—did indeed foster empathy toward their fellow students. Such activities are useful to complement other more direct methods of fostering empathy, such as asking students to write critical reflections from patients’ perspectives. Improvisation activities, by their very nature, are interactive, engaging, and may foster empathy in a more subtle fashion. That is, the feelings of empathy emerge as an implicit byproduct of the task, rather than being an explicit purpose of the task. The exercises were designed to require students to pay attention to the words and actions of their classmates and to participate actively in simple activities, which can make participants feel vulnerable and nervous, but to do so in a team and collegial atmosphere, which fosters support and a sense of teamwork. Being placed in this situation causes students to focus on their classmates, to evaluate both verbal and non-verbal communication modalities, and to create an environment in which they must actively work together to ensure any measure of success or completion. It is hypothesized that empathy is developed and deepened in this circumstance through a heightened requirement to truly listen, actively and presently, and forge a connection with classmates and teammates based on a sense of shared experience and creation.

More research is needed to further understand how such activities can be best incorporated into healthcare provider education, as the best practices for improvisation techniques regarding the content, duration, and frequency of sessions remain unclear.

## Figures and Tables

**Table 1:** Exploratory factor analysis results for the administration of revisions of the State Empathy Scale across the different cohorts and time points.

	Cohort 1 (*n* = 58)			Cohort 2 (*n* = 65)			Cohort 3 (*n* = 42)	
	Pre	Post	End of Semester	Pre	Post	End of Semester	Pre	Post
Item 1	0.48	0.32	0.38	0.74	0.58	0.70	0.30	0.63
Item 2	0.48	0.71	0.63	0.51	0.62	0.47	0.37	0.80
Item 3	0.62	0.70	0.50	0.50	0.47	0.59	0.32	0.74
Item 4	0.51	0.68	0.41	0.38	0.55	0.73	0.45	0.74
Item 5	0.76	0.71	0.64	0.51	0.65	0.62	0.31	0.61
Item 6	0.71	0.67	0.65	0.68	0.59	0.70	0.48	0.54
Item 7	0.74	0.58	0.63	0.63	0.64	0.60	0.42	0.58
Item 8	0.65	0.58	0.63	0.46	0.65	0.66	0.33	0.68
Item 9	0.67	0.58	0.44	0.40	0.52	0.54	0.70	0.56
Item 10	0.87	0.63	0.68	0.62	0.66	0.67	0.63	0.79
Item 11	0.82	0.69	0.74	0.69	0.56	0.77	0.69	0.77
Item 12	0.80	0.80	0.72	0.69	0.67	0.81	0.68	0.74
Eigenvalue	5.67	5.05	4.31	4.05	4.33	5.28	2.99	5.68
Average inter-item correlation	0.45	0.40	0.33	0.31	0.34	0.42	0.21	0.44
Cronbach’s Alpha	0.91	0.89	0.86	0.84	0.86	0.90	0.77	0.91

## Data Availability

All data are included in the paper.

## Appendix A

#	Please circle your program: PA MSBS					
Please respond to the following statements while imagining yourself communicating with students in the other program in this class. Please answer as honestly as possible, as there are no right or wrong answers.						
		Not at all				Completely
		0	1	2	3	4
1. My classmates’ emotions are		___	___	___	___	___
genuine.						
					
2. I can experience the same		___	___	___	___	___
emotions as my classmates						
when dealing with them.						
					
3. I am in a similar emotional		___	___	___	___	___
state as my classmates when						
dealing with them.						
					
4. I can feel my classmates’		___	___	___	___	___
emotions.						
					
5. I can see my classmates’		___	___	___	___	___
points of view.						
					
6. I recognize my classmates’		___	___	___	___	___
situation.						
					
7. I can understand what my		___	___	___	___	___
classmates are going through						
in class.						
					
8. My classmates’ reactions to		___	___	___	___	___
their situation are understandable.						
					
9. When dealing with my		___	___	___	___	___
classmates, I am fully absorbed.						
					
10. I can relate to what my		___	___	___	___	___
classmates are going through						
in class.						
					
11. I can identify with situations		___	___	___	___	___
described by my classmates.						
					
12. I can identify with my		___	___	___	___	___
classmates.						
						

## Appendix B

#						
Please respond to the following statements while imagining yourself communicating with students in the MSB program during this class today. Please answer as honestly as possible, as there are no right or wrong answers.						
		Not at all				Completely
		0	1	2	3	4
1. My classmates’ emotions are		___	___	___	___	___
genuine.						
					
2. I can experience the same		___	___	___	___	___
emotions as my classmates						
when dealing with them.						
					
3. I am in a similar emotional		___	___	___	___	___
state as my classmates when						
dealing with them.						
					
4. I can feel my classmates’		___	___	___	___	___
emotions.						
					
5. I can see my classmates’		___	___	___	___	___
points of view.						
					
6. I recognize my classmates’		___	___	___	___	___
situation.						
					
7. I can understand what my		___	___	___	___	___
classmates are going through						
in class.						
					
8. My classmates’ reactions to		___	___	___	___	___
their situation are understandable.						
					
9. When dealing with my		___	___	___	___	___
classmates, I am fully absorbed.						
					
10. I can relate to what my		___	___	___	___	___
classmates are going through						
in class.						
					
11. I can identify with situations		___	___	___	___	___
described by my classmates.						
					
12. I can identify with my		___	___	___	___	___
classmates.						
						

## Appendix C

**Table A1:** Mean and standard deviation (SD) values for all three cohorts of students at both or all three times of scale administration ^a^.

	Cohort 1 (*n* = 58)			Cohort 2 (*n* = 65)			Cohort 3 (*n* = 42)	
	Pre	Post	End of Semester	Pre	Post	End of Semester	Pre	Post
Mean	2.99	3.40	3.16	3.02	3.35	3.32	2.86	3.38
SD	0.60	0.45	0.46	0.46	0.42	0,44	0.46	0.52

^a^ All data sets represented in the three figures in the Results section passed normality tests (D’Agostino and Pearson test, Anderson–Darling test, Shapiro–Wilk test, and/or Kolmogorov–Smirnov test), except for cohort 1 in Figure 2A. Since parametric statistical analyses were not justified for the data in Figure 2A, nonparametric Friedman tests were used to draw similar statistical conclusions. While *p* < 0.0001 remained accurate for the difference between the Pre and Post data in Figure 2A, the Friedman test yielded *p* = 0.0002 for the difference between the Post and End of Semester data.

Moreover, the data in the table above should not be used for statistical analyses since they do not allow the pairing of data points for each individual student at the two or three separate times of scale administration. Differences in students’ self-reported empathy were tested by measuring students’ mean scores on a revised State Empathy Scale directly before versus directly after the improv session and comparing these means statistically using paired *t*-tests (cohort 3). When the scale was administered a third time at the end of the semester (student cohorts 1 and 2), the mean scores were also compared statistically using one-way analysis of variance combined with multiple comparison tests of score values matched for each student. When Friedman tests were used for the statistical analyses, the score values were still matched for each student.

## References

[1] Nembhard I.M., David G., Ezzeddine I., Betts D., Radin J. (2023). A systematic review of research on empathy in health care. Health Serv. Res..

[2] Watts E., Patel H., Kostov A., Kim J., Elkbuli A. (2023). The role of compassionate care in Medicine: Toward improving patients’ quality of care and satisfaction. J. Surg. Res..

[3] Van Winkle L.J., Burdick P., Bjork B.C., Chandar N., Green J.M., Lynch S.M., La Salle S., Viselli S.M., Robson C. (2014). Critical thinking and reflection on community service for a medical biochemistry course raise students’ empathy, patient-centered orientation, and examination scores. Med. Sci. Educ..

[4] Schwartz B.D., Horst A., Fisher J.A., Michels N., Van Winkle L.J. (2020). Fostering empathy, implicit bias mitigation, and compassionate behavior in a medical humanities course. Int. J. Environ. Res. Public Health.

[5] Hojat M. (2016). Empathy in Health Professions Education and Patient Care.

[6] Starcevic V., Piontek C.M. (1997). Empathic understanding revisited: Conceptualization, controversies, and limitations. Am. J. Psychother..

[7] Hojat M., Mangione S., Kane G.C., Gonnella J.S. (2005). Relationships between scores of the Jefferson scale of physician empathy (JSPE) and the interpersonal reactivity index (IRI). Med. Teach..

[8] Shen L. (2010). On a scale of state empathy during message processing. West. J. Commun..

[9] Zhang X., Pang H.F., Duan Z. (2023). Educational efficacy of medical humanities in empathy of medical students and healthcare professionals: A systematic review and meta-analysis. BMC Med. Educ..

[10] Lu N., Ma Z., Shi Y., Yao S., Zhang L., Shan J., Zhai L., Li C., Cheng F. (2023). A narrative medicine-based training program increases the humanistic care quality of new nurses in cancer hospital. Precis. Med. Sci..

[11] Zhao J., Xiantao O., Li Q., Liu H., Wang F., Li Q., Xu Z., Ji S., Yue S. (2023). Role of narrative medicine-based education in cultivating empathy in residents. BMC Med. Educ..

[12] Omer S., McCarthy G. (2010). Reflective practice in psychiatric training: Balint groups. Ir. J. Psychol. Med..

[13] Silva J.A.C.D., Massih C.G.P.A., Valente D.A., Souza D.F.D., Monteiro M.R.L.D.C., Rodrigues R.M. (2023). Teaching empathy in healthcare: An integrative review. Rev. Bioética.

[14] Kaplan-Liss E., Lantz-Gefroh V., Bass E., Killebrew D., Ponzio N.M., Savi C., O’Connell C. (2018). Teaching medical students to communicate with empathy and clarity using improvisation. Acad. Med..

[15] Zelenski A.B., Saldivar N., Park L.S., Schoenleber V., Osman F., Kraemer S. (2020). Interprofessional improv: Using theater techniques to teach health professions students empathy in teams. Acad. Med..

[16] O’Connell C., McCauley J., Herbert L. (2022). Improvisation-Based Workshop to Build Empathy in Mentor-Mentee Relationships and Support Academic Equity. J. Stud. Aff. Res. Pract..

[17] Alda A. (2018). If I Understood You, Would I Have This Look on My Face?. My Adventures in the Art and Science of Relating and Communicating.

[18] Schmidt I., Rutanen T., Luciani R.S., Jola C. (2021). Feeling for the other with ease: Prospective actors show high levels of emotion recognition and report above average empathic concern, but do not experience strong distress. Front. Psychol..

[19] Acock A.C. (2014). A Gentle Introduction to Stata.

[20] Hojat M., Xu G. (2004). A visitor’s guide to effect sizes–statistical significance versus practical (clinical) importance of research findings. Adv. Health Sci. Educ..

[21] Rosenthal R., Rubin D.B. (1982). A simple, general purpose display of magnitude of experimental effect. J. Educ. Psychol..

[22] Adamson K., Loomis C., Cadell S., Verweel L.C. (2018). Interprofessional empathy: A four-stage model for a new understanding of teamwork. J. Interprof. Care.

[23] Michalec B., Schneider J.M., Mackenzie M. (2021). Teaching empathy in an interprofessional setting with a focus on decategorization: Introducing I-Team. J. Interprof. Educ. Pract..

[24] Van Winkle L.J., Fjortoft N., Hojat M. (2012). Impact of a workshop about aging on the empathy scores of pharmacy and medical students. Am. J. Pharm. Educ..

[25] Baguley S., Dev V., Fernando A.T., Consedine N.S. (2020). How do health professionals maintain compassion over time? Insights from a study of compassion in health. Front. Psychol..

[26] Sinclair S., Harris D., Kondejewski J., Roze des Ordons A.L., Jaggi P., Hack T.F. (2023). Program leaders’ and educators’ perspectives on the factors impacting the implementation and sustainment of compassion training programs: A qualitative study. Teach. Learn. Med..

[27] Sinclair S., Kondejewski J., Jaggi P., Roze des Ordons A.L., Kassam A., Hayden K.A., Harris D., Hack T.F. (2021). What works for whom in compassion training programs offered to practicing healthcare providers: A realist review. BMC Med. Educ..

[28] Martimianakis M.A.T., Michalec B., Lam J., Cartmill C., Taylor J.S., Hafferty F.W. (2015). Humanism, the hidden curriculum, and educational reform: A scoping review and thematic analysis. Acad. Med..

[29] Sharma M., Pinto A.D., Kumagai A.K. (2018). Teaching the social determinants of health: A path to equity or a road to nowhere?. Acad. Med..

[30] Mercer S.W., Maxwell M., Heaney D., Watt G.C. (2004). The consultation and relational empathy (CARE) measure: Development and preliminary validation and reliability of an empathy-based consultation process measure. Fam. Pract..

